# Conference highlights of the 15th international conference on human retrovirology: HTLV and related retroviruses, 4-8 june 2011, Leuven, Gembloux, Belgium

**DOI:** 10.1186/1742-4690-8-86

**Published:** 2011-10-28

**Authors:** Fabiola Martin, Charles RM Bangham, Vincenzo Ciminale, Michael D Lairmore, Edward L Murphy, William M Switzer, Renaud Mahieux

**Affiliations:** 1 Centre for Immunology and Infection, Department of Biology, Hull and York Medical School, University of York, York, UK; 2 Department of Immunology, Wright-Fleming Institute, Imperial College London, London, UK; 3 Department of Oncology and Surgical Sciences and Istituto Oncologico Veneto-Istituto di Ricovero e Cura a Carattere Scientifico (IRCCS), Padova, Italy; 4 Department of Veterinary Biosciences; Centre for Retrovirus Research; and Comprehensive Cancer Centre, The Arthur James Cancer Hospital and Research Institute, The Ohio State University, Columbus, Ohio, USA; 5 University of California San Francisco and Blood Systems Research Institute, San Francisco, California, USA; 6 Laboratory Branch, Division of HIV/AIDS Prevention, National Centre for HIV/AIDS, Viral Hepatitis, STD, and TB Prevention, Centres for Disease Control and Prevention, Atlanta, GA 30333, USA; 7 Retroviral Oncogenesis Laboratory, INSERM-U758 Human Virology, 69364 Lyon cedex 07, France; 8 Ecole Normale Supérieure de Lyon, 69364 Lyon cedex 07, France

## Abstract

The June 2011 15^th ^International Conference on Human Retrovirology: HTLV and Related Viruses marks approximately 30 years since the discovery of HTLV-1. As anticipated, a large number of abstracts were submitted and presented by scientists, new and old to the field of retrovirology, from all five continents. The aim of this review is to distribute the scientific highlights of the presentations as analysed and represented by experts in specific fields of epidemiology, clinical research, immunology, animal models, molecular and cellular biology, and virology.

## Introduction

The biannual Conference on Human Retrovirology: HTLV and Related Viruses, where 325 delegated from 17 countries gathered, was held in Leuven, Belgium in June 2011. Two hundred and sixty one abstracts were submitted, and those accepted divided into 77 oral and 184 poster presentations. All abstracts can be viewed online: http://htlv.net and http://www.retrovirology.com/supplements. Experts in seven fields of retrovirology were asked to write summaries and comments on the most intriguing novel data and share their views on future research directions. During the meeting, Professor K.T. Jeang received the The Dale McFarlin Prize, Professor L. Willems was awarded with the HTLV Retrovirology Prize and the Young Scientists Awards went to A. Desrames, J. Turpin and C. Hhela.

The human T-lymphotropic virus type 1 (HTLV-1) retrovirus infects 15 to 20 millions individuals throughout the world. HTLV-1 antibody prevalence rate varies from 0.2 to 10% among adults, depending on the geographical area. It increases with age, in some places eventually reaching 20 to 50% of the female population aged 60 and above. The two major diseases associated with HTLV-1 are Adult T-cell Leukemia/Lymphoma or ATLL and HTLV-1 Associated Myelopathy/Tropical Spastic Paraparesis or HAM/TSP [1]. Xenotropic Murine Leukemia virus-Related Virus (XMRV) was identified in 2006 in stromal cells associated with prostate cancers [2] and later in patients suffering from chronic fatigue syndrome. The etiological role of XMRV in these two diseases has recently been challenged. Bovine Leukemia virus (BLV) infects B-lymphocytes and cause B leukemia [3]. Foamy viruses infect a wide number of animal species, as well as humans, but do not cause any disease [4].

## Epidemiology

### Epidemiology of HTLV

Two studies used the blood bank setting to measure HTLV prevalence on a large scale. A study from the United States of America [5] included all first-time blood donors at a large blood bank network over a ten-year period from 2000 through 2009. Among a total of 1,904,155 first-time blood donors, confirmed HTLV-1 and -2 prevalence was 1.43 per 10^4 ^(95% CI 1.19-1.72), including HTLV-1 0.40 per 10^4^; HTLV-2 0.87 per 10^4^; and HTLV untypeable 0.25 per 10^4^. HTLV-1/2 prevalence decreased from 2000 to 2009 (p trend < 0.0001), consistent with a birth cohort effect for HTLV infection in the United States of America as also described in Japan. *Proietti et al*. performed a similar analysis in Brazil for 2007-2009 among 281,760 first-time blood donors [6]. Overall HTLV-1 prevalence was 12.9 per 10^4 ^(95% CI 11.6-14.2), with regional variation, but little changed over the three years of the study. The same study calculated the HTLV-1 incidence among blood donors in Brazil at 3.6 per 10^5 ^person-years, leading to an estimated residual transfusion risk after current blood bank testing of 5.0 per 10^6 ^blood units transfused, about 10 times higher than in the United States of America [7]. HTLV prevalence data from Africa are rare, but *Caterino-de-Araujo et al*., studied several hundred patients attending public health clinics in northern, central and southern Mozambique, respectively [8]. An overall HTLV-1/2 prevalence of 2.3% was observed, with regional variation; one HTLV-1 isolate was sequenced as the Cosmopolitan subtype.

### Molecular epidemiology of HTLV

In the STLV section, below, William Switzer has covered some interesting new findings on HTLV-2 molecular epidemiology in the Cameroonian rainforest. Those data confirmed the close sequence homology between HTLV-2 in Bakola Pygmies with that found in South American Indians, consistent with ancient human migrations. Changing continents, *Desrames et al*. analyzed LTR sequences from 55 HTLV-1 carriers from the Caribbean and Africa. They found that all of the West Indian strains except one belong to the Cosmopolitan subtype, and only the Noir Marron ethnic group from French Guiana was infected by the West African strain [9]. *Magri et al*. analyzed samples from Brazil and found concordance with HTLV-1 isolates from Angola and Mozambique. These data suggested HTLV molecular studies can shed new light on historical information regarding slavery-related human transport and the transmission of HTLV-1 from Africa to the New World.

### Disease associations and pathogenesis

A multicenter collaborative investigation of biomarkers which might predict ATL was reported by *Birmann et al*. [10]. A total of 53 incident ATL cases and 150 carefully matched asymptomatic HTLV-1 carriers were obtained from population-based studies in Japan, Jamaica, the United States of America, and Brazil, and multiple biomarkers were measured on PBMCs and serum. Elevated levels of soluble interleukin-2 receptor alpha (sIL2Rα) and anti-Tax antibody were both significantly associated with ATL. Elevated levels of total immunoglobulin E (IgE) predicted a lower risk of ATL. The authors suggested that a biomarker pattern predictive of heightened T-cell activation and HTLV-1 replication, as well as diminished type II immunity, can be associated with ATL.

*Izumo et al*. reported on a large national survey of HAM/TSP across 829 neurology clinics in Japan during 2007 and 2008 [11]. Response rate was 33.5%; and 790 cases of HAM/TSP were reported, leading to a national estimate of 3600 HAM/TSP cases; half of the patients lived in Kyushu and 15% each lived in the Tokyo and Osaka metropolitan areas. *Costa et al*. reported on the neurological manifestations of 102 HTLV-1 patients without HAM/TSP [12]. The prevalence of neurologic symptoms as well as arm and leg weakness on examination was significantly associated with overactive bladder after adjusting for age and sex. Two studies examined neuropsychiatric outcomes of HTLV infection. *Guiltinan et al*. found no association between HTLV-1 or HTLV-2 and depression or generalized anxiety in a prospective cohort study of former blood donors, after adjusting for education, alcohol intake and self-reported health status [13]. Poor health status early in the study, as measured by the general well-being scale, predicted depression and anxiety about 15 years later. In an uncontrolled study, *Galvao-Phileto et al*. found a 35% prevalence of depression among persons living with HTLV-1 infection in Salvador de Bahia, Brazil. Depression was associated with reporting poor quality of life [14]. Taken together, these studies suggest that HTLV related health problems and social environment, rather than viral infection alone, lead to psychiatric symptoms.

*Arruda et al*. performed a meta-analysis of the association between HTLV infection and active tuberculosis and yielded an estimated relative risk of 3.25 for the association between HTLV infection and active tuberculosis [15]. The author suggests that tuberculosis chemoprophylaxis might be considered in HTLV-1 carriers living in tuberculosis endemic areas. *Einsiedel et al*. reported an association between HTLV-I infection and bronchiectasis and pulmonary mortality among Australian aborigines [16].

### Future directions

Ongoing, large-scale surveillance of HTLV prevalence is needed in endemic countries, and blood donor data provide an inexpensive and largely comparable way of doing this across countries. Molecular epidemiologic studies may be increasingly useful in identifying fine scale patterns of HTLV dissemination, but would be even more useful if they incorporated parallel data on human genetics to complement viral sequence data. Especially in endemic countries, prospective cohort studies are needed to better define prognostic markers for HTLV diseases.

## Clinical Research

### Clinical Presentations

For the last 30 years, clinicians and scientists have been searching for a disease dependent diagnostic, disease progression predictive, and therapy responsive biomarker in HTLV-1 infection, similar to HIV-1 serum RNA and CD4^+ ^T cell count. So far we know that high HTLV-1 proviral load/100 PBMCs (≥ 10%), high CD4^+^/CD25^+ ^T cell lymphocyte count, and oligoclonal expansion are associated with HTLV-1 associated disease, such as HTLV-1 associated myelopathy and ATL.

Similarly *Demontis et al*. reported that 15% of asymptomatic carriers (AC) attending the National Centre for Human Retrovirology in London, UK, had a proviral load (pVL) > 10% PBMCs, and 3% even had a pVL > 20% PBMCs [17]. These carriers might need long term, close surveillance. She also reported a high mean intra-patient variability of pVL, not only in AC (65%, SD 21), but also in patients with HAM/TSP (51%, SD 23). If reproduced, this must be taken into consideration for any HTLV-1 clinical trial sample size calculation.

The association between HTLV-1 pVL and HAM/TSP was confirmed by *Grassi et al*. but the proviral load did not differentiate between the three Belem diagnostic criteria of HAM/TSP (possible, probable and definite) [18]. *Yamano et al*. reported high levels of CXCL10 (Interferon γ-induced protein) in cerebrospinal fluid (CSF) and serum, neopterin in CSF, and soluble IL-2 receptor in serum to correlate with severity of HAM/TSP disease (n = 30). They also classified patients into different stages of HAM/TSP disease [19]. This approach would allow the recruitment of patients with similar disease severity into clinical trials.

### Treatments for HAM/TSP

*Gotuzzo et al*. observed that routine treatment of patients with rapidly progressive HAM/TSP (< 2 years onset) with oral prednisolone together with lamivudine +/- zidovudine for 12 months led to a significant decrease in HTLV-1 pVL in 7/11 patients (p = 0.01) and an insignificant increase in 4/11 patients (p = 0.06) at the 7 months follow up [20]. An early, but only transient clinical improvement, was observed (personal communication). Only a randomised control trial will be able to control for the natural fluctuation of pVL within patients and show true treatment effect.

Raltegravir, an integrase inhibitor, is a licensed antiviral drug against HIV and also inhibits cell free and cell associated transmission of HTLV-1 as well as the immortalisation of HTLV-1 positive PBMCs *ex vivo *[21]. Contrary to previous reports, *Fox et al*. detected 1 and 2 episomal long terminal repeat (LTR) HTLV-1 DNA circles in freshly and chronically HTLV-1 infected cell lines and patient PBMCs (n = 16). These were readily detected in patients with HAM/TSP and ATL, but not in AC. A significant rise in 2LTR DNA circles was observed in freshly infected and raltegravir treated CEM cell lines, especially within the first three days of infection [22]. *In vivo*, however, *Trevino et al*. observed only a transient decrease of HTLV-1 pVL in two HAM/TSP out of five HTLV-1 positive patients and no clinical improvement, when treated for 12 months with raltegravir mono-therapy [23].

### Treatments for ATLL

Haematopoetic stem cell transplantation (HSCT) remains so far the only curative intervention for ATL albeit with high myeloablation associated mortality. *Uike et al*. reported the long term survival of 9/10 patients with ATL treated in the NST-1/NST-2 clinical trial (HSCT + reduced intensity conditioning) regime [24] at a median 115 months follow up. All patients showed complete donor chimera and 3/10 patients had undetectable HTLV-1 pVL at the last follow up [25]. Relapse of ATL is frequent and difficult to treat, despite the modernisation of first line chemotherapy with additional azidothymidine (AZT) and interferon (IFN). *Suarez et al*. treated patient with ATL with arsenic trioxide + IFN α combination as maintenance therapy following induction chemotherapy +/- AZT and IFN α. Five/11 patients (4 CR, 1 PR) were alive at follow up (9 to 46 months) of whom 3/5 patients had lymphomatous ATL. However 6/11 patients who were progressing at the time of maintenance therapy did not respond and died [26]. Larger randomised controlled phase III trials are needed to reduce this selection bias.

Following a phase I trial [27], *Utsunomiya et al*. treated 13 patients with CCR4+ relapsed ATL with anti-CCR4 antibody (KW-0761, i.v. 1 mg once weekly for 8 weeks) in multicentre phase II trial. The overall response in 26/28 patients was 50% (8 CR, 5PR). Notably there was a marked difference between median overall and progression free survival (13.7 vs. 5.2 months) [28].

HTLV-1 persists by driving clonal proliferation of infected T lymphocytes [29], and several studies tested the reinstatement of polyclonal versus oligoclonal T lymphocytes as a marker of treatment response. *Ramos et al*. presented preliminary clinical trial data of adding histone deacetylase (HDAC) inhibitor valproic acid to maintenance therapy AZT+INF-α in 13 patients with acute ATL and observed a serial decrease in clonal ATL disease followed by molecular clearance in one patient. *In vitro *testing of newer, more potent HDAC inhibitors are planned [30]. *Hodson et al*. observed a 33% response rate in acute ATL (n = 3, median OS: 3 months, 1CR) and 100% in chronic ATL (n = 5, median OS: 20 months) to AZT+ INF-α as first line therapy alone [31]. Again, not only a reduction in pVL, but also the re-emergence of polyclonal integration patterns were associated with disease remission.

### Future directions

In summary, randomised controlled trials remain urgently needed to determine reliable biomarkers of HTLV-1 disease and of treatment response as well as to establish a treatment strategy especially for lymphomatous ATL, relapsed ATL and any stage of HAM/TSP.

## Animal Models

Presentations during the Animal Models section illustrate ongoing efforts to understand the pathogenesis of HTLV-1-associated diseases using a variety of model systems. The development of accurate and reproducible animal models is critical to the understanding of the pathogenesis of HTLV-1-associated diseases. No perfect model exists that recapitulates all aspects of HTLV-1 diseases syndromes. Transmission and viral spread of HTLV-1 have been studied in rabbits and non-human primates, but lesion development and reagents are limited in these species. As reflected in the meeting abstracts, the mouse provides a cost effective and highly reproducible model in which to study factors related to lymphoma development and the preclinical efficacy of potential therapies against adult T-cell lymphoma and leukemia (ATL). These include important transgenic mouse models that have been utilized to study viral determinants of lymphocyte transformation in vivo.

### Immunodeficient mice

The six presentations chosen for platform presentations covered a variety of relevant topics. Xenografts of adult T-cell leukemia/lymphoma (ATL) cells or cell lines into immunodeficient mice replicate features of ATL and allow systems to test therapies against the neoplastic disease [32]. *Tezuka et al*. reported their development of ATL-like disease in humanized mice (huNOG) by the intra-bone marrow transplantation of NOG-SCID mouse with CD133^+ ^hematopoietic stem cells purified from human cord blood infected with HTLV-1 [33]. Inverse PCR analysis of provirus integration sites revealed oligoclonal expansion of infected T cells in CD4^+^/CD25^+ ^T-cells similar to HTLV-1-infected humans. *Villaudy et al*. reported that HTLV-1 induces alterations of the thymus of Rag2^-^/IL-2R γc^- ^mice leading to expanded populations of mature CD4^+^/CD25^+ ^T cells and other pathological features such as splenomegaly and lymphomas compared with mock-infected mice [34,35]. This unique model system was then used to test anti-cancer drugs in a related abstract, further illustrating the usefulness of the model. *Van den Broeke et al*. [36] utilized NOD-Scid-γ immunodeficient mice to test *in vivo *kinome profiles from BLV-induced leukemic sheep.

### Transgenic mice

Transgenic mouse models continue to provide new insights into the molecular mechanisms of HTLV-1 Tax. *Swaims et al*. reported on the role of HTLV-1 expression in chronically-infected CD4^+ ^T cells using LTR-Tax Transgenic mice [37]. In this system, immune activated Tax CD4^+ ^T cells express characteristics of several different CD4^+ ^T cell subtypes, suggesting that HTLV-1 Tax induces changes in the normal pattern of CD4^+ ^subtype specification. In an interesting study using a bioinformatics approach, *Suzuki et al*. [38] identified proteins differentially expressed in a model of Tax-induced lymphoma [39]. Among the more than 700 proteins detected, levels of 53 proteins were increased in stem cells, including one membrane protein, which might potentially serve as a new target of antibody-based therapy.

*Shinagawa et al*. [40] constructed a Transgenic (Tg) rat expressing human CRM1 (hCRM1), a cellular cofactor of Rex, and provided data that T cells derived from Tg rats allowed production of HTLV-1 as efficiently as human T cells [41]. Their results suggest the presence of inhibitor(s) during the entry process in rat dentritic cells. *Rosewick et al*. [42] reported ongoing studies in the bovine leukemia virus (BLV) model using high-throughput sequencing to reveal down-regulation of small non-coding viral RNAs that might contribute to tumor-associated virus silencing and play a role in immune escape mechanisms [43]. *Ohsugi and Kumasaka *reported their findings of a transgenic mouse model of arthropathy expressing Tax in mature thymocytes and peripheral T lymphocytes [44,45]. *Rauch et al*. reported that Tax expression in IL-15 knockout mice [46,47] led to the development of larger and more aggressive tumors, suggesting caution against IL-15 blockade as an ATLL therapy [8]. *Taguchi et al*. [48] investigated the production of cytokines in HBZ Transgenic (HBZ-Tg) mice and provided data to support the concept that altered Foxp3 expression in iTreg cells may result in systemic inflammation [49].

### Nonhuman primates

*Moura et al*. provided promising data for new targeted therapy against ATL in a monkey model [50]. They demonstrated that HTLV-1-infected cells constitutively express high levels of surface transferrin receptor (TfR) in acute forms of ATL and tested a monoclonal antibody (mAb A24) on ATL cells *ex vivo*. Importantly, in squirrel monkeys, (Saimiri sciureus), the administration of single and repeated doses of A24 did not induce significant toxicity yet increased transferrin and elicited apoptosis in lymph node samples in areas of high lymphocyte proliferation (germinal centres). This new agent (A24) is a potential new treatment of acute forms of ATL.

### Rabbits

*Haines et al*. provided data on the development of an oral model of HTLV-1 transmission in rabbits to allow testing of the mucosal microenvironment during the early stages of orally-acquired HTLV-1 [51].

### BLV studies

Several investigators provided data on host and viral determinants of BLV infection. *Gutiérrez et al*. [52] provided intriguing data on the efficacy of a life-attenuated BLV vaccine to prevent viral transmission in cattle [53]. *Aida et al*. in a highly collaborative project identified genetic markers of BLV induced disease [54].

### Future directions

There is a great need to continue developing models that reflect all stages of the infection and the full range of disease syndromes in humans infected with HTLV-1. Important gaps exist in the knowledge of early mucosal transmission and cell-to-cell transmission, such as early target cells and the tissue environmental influences that allow the virus to spread following oral and sexual routes of transmission. In addition, few animal models accurately mimic the neurologic disorders associated with HTLV-1 infection. Finally, models to reproduce the influence of co-infections with HTLV-1 and how these agents exacerbate the spectrum of HTLV-1 disease are needed to fully understand the pathogenesis of HTLV-1 in humans and to assist in the development of new therapeutic agents to ablate these devastating diseases.

## Molecular and cellular biology

### Regulation of HTLV-1 5'LTR

The primary activity of Tax is to modulate viral expression through the CREB/ATF pathway. Using HTLV-1 integrated chromatin templates, *Easley et al*. showed that an active viral promoter uses the chromatin remodelling complex PBAF [55]. *McCabe et al*. suggested that the cellular FHL3 protein is involved in Tax-mediated transactivation. On the other hand *Robette et al*. [56] described that in latently infected cells, but not in cells that actively produce HTLV-1 virions, the CTIP2 complex (which contains histone deacetylases and a methyltransferase) is recruited onto the 5' LTR and inhibits Tax-mediated transcription, similar to what was described for HIV-1 [57].

### Tax post-translational modifications and NF-κB activation

Tax is also involved in cell transformation, notably through the constitutive activation of the NF-κB pathway that can be divided into cytoplasmic and nuclear events. Ubiquitinated Tax is mostly found in the cytoplasm, while SUMOylated Tax is located in the nucleus. However, how Tax precisely shuttles between the cytoplasm and the nucleus and which fractions play a role in the NF-κB pathway is still a matter of debate. *Kfoury et al*. demonstrated that the same Tax molecule shuttles between the cytoplasm and the nucleus [58,59]. Ubiquitination targets Tax to nuclear bodies, but interestingly both ubiquitination and SUMOylation control IKKγ targeting to the centrosome where NF-κB activation begins. *Bex et al*. demonstrated that Tax co-localizes with TAB2- and IKKγ-positive structures in the cytoplasm, suggesting that they represent an important domain in the NF-κB activation process [60]. Whether the Tax species that are present there are ubiquitinated or not was not determined. *Yasunaga et al*. showed that the ubiquitinated fraction of Tax is a substrate of USP20, an ubiquitin specific peptidase, leading to Tax deubiquitination and to the suppression of NF-κB activation [61,62]. Interestingly, USP20 levels are low in HTLV-1-infected cells, where NF-κB is usually elevated. It would now be of interest to determine whether this protein promotes the accumulation of SUMOylated Tax. By contrast, *Lavorgna et al*. described that STAMBPL1, another deubiquitinating (DUB) enzyme, is necessary for Tax activity [63]. Importantly, STAMBPL1 does not induce Tax de-ubiquitination but protects Tax from degradation. The target of STAMBPL1 now remains to be identified. *Fryrear et al*. showed that the SUMOylated nuclear fraction is a substrate of RNF4, a SUMO-targeted ubiquitin ligase (STUbL) [64]. This promotes Tax translocation from nucleus to cytoplasm and its ubiquitination. siRNA suppression of RNF4 prevented damage-mediated nuclear egress of Tax. Interestingly, using T lymphocytes, *Bonnet et al*. showed that a Tax mutant that is ubiquitinated, but not SUMOylated can reach the nucleus, but is severely impaired for NF-κB activation, underscoring the complexity of the relationship between localization, post-translational modification and transcriptional activity of Tax [65].

**Figure 1:**
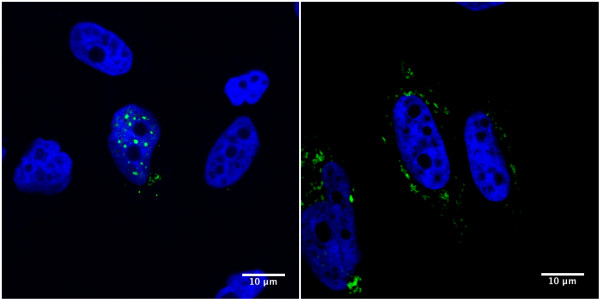
**HTLV-1 Tax (Tax1) and HTLV-2 Tax (Tax2) have different subcellular localization**. pSG5M-Tax-1-His (left) and pSG5M-Tax-2B-His (right) were transiently transfected into coverglasses-seeded HeLa cells. Twenty four hours post-transfection, cells were fixed and stained with a rabbit anti-histidine antibody (sc-803, Santa Cruz Biotechnology) followed by a fluorescein-conjugated goat anti-rabbit antibody (**green**, Vector). Coverglasses were then mounted with DAPI-containing mounting medium (**blue**, Fluoromount-G, Southern Biotech) and cells were visualized using a Leica TCS SP5 confocal microscope. Images of cells that are representative of the entire population are shown.

Adding to the complexity, *Journo et al*. demonstrated that the HTLV-2 Tax protein, which does not localize like HTLV-1 Tax in the cell (Figure 1), is barely ubiquitinated [66]. Nevertheless, it efficiently recruits IKKγ, promotes RelA nuclear translocation, and leads to NF-κB activation. *Turci et al*. showed however that Tax2 can be ubiquitinated and SUMOylated under experimental conditions where ubiquitin or SUMO is overexpressed [67]. *Shembade et al*. demonstrated that Tax prevents TAX1BP1 phosphorylation and therefore allows a constitutive NF-κB activation [68]. *Fu et al*. also showed that both the canonical and the non-canonical NF-κB pathways play roles during Tax-mediated tumorigenesis *in vivo *[69]. Even if NF-κB is activated, Tax expression is usually low in leukemic cells. *Yamagishi et al*. showed that mir-31 is down regulated in these cells. Interestingly, a target of mir-31 is NIK, a kinase that is involved in NF-κB activation [70]. Altogether, these results suggest that (i) both HTLV-1 Tax ubiquitination and SUMOylation are important for the activation of the NF-κB pathway, (ii) several DUBs are directly or indirectly involved in the regulation of this process, (iii) HTLV-2 Tax does not function as its HTLV-1 counterpart, and (iv) in ATL cells, the NF-κB pathway is possibly activated through the down-regulation of cellular miRNAs.

### HBZ and cellular transformation

Expression of Tax is rarely detected in ATL cells, thus favoring a model in which Tax is necessary to initiate cell immortalisation and transformation, but not the proliferation of the tumour cells. The HBZ viral protein might play a major role at the late stage of the disease, by preventing transcription from the 5' LTR and therefore *tax *expression, but also by promoting cell proliferation. *Wright et al*. showed that, in addition to its physical interaction with CBP/p300 proteins, HBZ inhibits their Histone Acetyl Transferase (HAT) activity and consequently represses 5'LTR transcription. Inhibition of CBP/p300 HAT function also leads to a decreased activity of other cellular transcription factors such as p53 or p65/RelA [71]. It would now be important to determine the fate of these cells, where CBP/p300 functions are impaired. *Peloponese et al*. determined how the HBZ promoter is regulated and suggested that, in addition to Tax, HBZ itself regulates its own promoter through its interaction with JunD and its action on the Sp1 binding sites [72]. Working on viral latency and viral expression, *Choudhary et al*. demonstrated that HBZ expression results in a lower *p30 *mRNA expression [73,74]. p30 is a negative post-transcriptional regulator. It specifically reduces the level of *tax/rex *cytoplasmic *mRNAs *in a dose-dependent manner resulting in a decreased viral replication. This was demonstrated both in cells transfected with an HTLV-1 molecular clone and in HTLV-1 infected cells transduced with VSV-p30 pseudotyped viruses [75]. These results would suggest that HBZ indirectly favours Tax expression. Consistent with previous reports demonstrating that HBZ promotes cell proliferation, *Mukai & Ohshima *suggested that HBZ activates the mTOR signalling which plays a role in cell proliferation [76], while *Zhi et al*. reported that HBZ down-regulates NF-κB and prevents Tax-mediated senescence, therefore allowing the infected cells to divide [77,78]. Given that ATL cells display a constitutively active NF-κB pathway, these results suggest that the negative effect of HBZ on the NF-κB pathway is counteracted, possibly through mir-31 down-regulation.

*Marban et al*. demonstrated that APH-2 (the HTLV-2 counterpart of HBZ) binds c-Jun, but contrary to HBZ, enhances its ability to activate AP-1 [79]. Interestingly, *Larocque et al*. showed that the APH-3 and APH-4 proteins (encoded respectively by HTLV-3 and HTLV-4) also activate JunB, c-Jun and JunD-mediated transcription [80]. Altogether, these results suggest that, apart from HBZ, and despite having very divergent amino-acid sequences, all APH proteins activate AP-1 factors. Whether this plays a role *in vivo *remains to be determined.

### HTLV auxiliary proteins

The p12, p13, p30 proteins play an important role in the HTLV-1 viral cycle and in viral persistence *in vivo. Edwards et al*. showed that following palmitoylation and localization to membrane lipid rafts, both p12 and its cleavage product p8 can form homo- and hetero-dimers [81]. Interestingly, some HTLV-1 infected individuals carry a virus encoding a mutated, dimerisation-impaired p8 protein. Because p8 is involved in viral transmission, this could suggest that some patients are better transmitters than others. *Silic-Benussi et al*. demonstrated that p13 increased mitochondrial ROS production resulting in activation of primary T-cells and sensitization to death of tumour T- cells [82,83]. *Andresen et al*. suggested that, in the presence of Tax, p13 partially co-localizes and directly binds Tax in nuclear speckles. It decreases Tax binding to the CBP/p300 transcriptional co-activator, and, by reducing Tax transcriptional activity, suppresses viral expression [84,85]. This suggests that p13 also negatively regulates the viral expression and therefore favours latency. Finally, *Turpin et al*. demonstrated the existence of two spliced mRNAs encoded by STLV-3 pX ORFs. One of these mRNA encodes a protein (tentatively named p8) whose function seems similar to that of the HTLV-1 p30 or HTLV-2 p28, although its precise mechanism of action remains unknown [86].

### Future directions

It appears now clearly that Tax's post-translational modifications impact its cellular localization, either in the cytoplasm or in the cell nucleus and therefore its function. Despite a number of recent findings a number of questions still exist: i) what is the exact role of Tax post-translational modifications in NF-κB activation and in LTR transcription? ii) How are these post-translational modifications regulated? How is one Tax species converted to another? iii) Since at least three different HTLV-1 proteins repress Tax expression, how exactly is the HTLV-1 cycle regulated in vivo? More research needs to be done into the intimate interplay between Tax and HBZ and their consequence on cell cycle, NF-κB and HTLV-1 pathogenesis.

## Virology

### Viral transmission

HTLV-1 propagation and persistence *in vivo *depend on both *de novo *entry into host cells ("infectious transmission") and "mitotic transmission" of the integrated viral genome to daughter cells. Studies on HTLV-1 entry focused on the importance of dendritic cells [87,88] and on the HTLV-1 binding and entry into cells, which involve the hierarchical interaction of viral envelope glycoproteins with three molecules on the surface of target cells: heparan sulfate proteoglycans, Neuropilin 1 and the glucose transporter GLUT1 [89]. *Jensen et al*. also showed evidence for a role in HTLV-1 entry of Xylosyltransferase 2, an enzyme encoded on chromosome 17q [90]. Efficient cell-to-cell spread of HTLV-1 occurs via a highly organized cell-cell contact known as the virological synapse (VS). *Nejmeddine et al*. showed exclusion of actin microfilaments in the VS in infected T-cells, a change that may facilitate viral transmission [91]. Mother-to-child transmission through breastfeeding is a major mode of HTLV-1 transmission. *Martin-Latil et al*. developed an *in vitro *model to study passage of HTLV-1 through an epithelial barrier. Results showed that, although enterocytes were not susceptible to infection, HTLV-1 virions were detected in the basal compartment, suggesting a transcytotic mechanism of passage [92].

### Dynamics of HTLV-1 propagation/expression in vivo

*Gillet et al*. demonstrated that negative selection (possibly exerted by CTLs) dominates during chronic infection, favouring proviruses integrated in transcriptionally silenced DNA [93].

Using splice site-specific qRT-PCR, *Rende et al*. demonstrated a two-phase kinetics of HTLV-1 gene expression in PBMCs from infected patients, with the expression of Tax/Rex mRNA preceding that of other viral transcripts; the authors also demonstrated the Rex-dependency of this kinetics and showed that over 90% of the HBZ mRNAs were localized in the nucleus [94]. *Bender et al*. showed that HTLV-2 exhibits a similar 2-phase kinetics. However, a distinguishing characteristic of HTLV-2 was its higher expression of mRNAs encoding potential inhibitors of Tax and Rex, i.e. p28 and truncated isoforms of Rex [95]. Future studies should be aimed at testing whether HTLV-2 exhibits a more latent pattern of expression compared to HTLV-1. *Belrose et al*. also analyzed HTLV-1 expression in PBMCsfrom infected patients and showed that the HDAC inhibitor valproate enhanced Tax while it blocked HBZ expression, suggesting that the balance between Tax and HBZ may determine the outcome of VPA treatment [96]. *Oka T et al*. reported that the number of genes with methylated CpG islands increased with ATLL progression, especially in ATLL lymphomas. The high number of methylated genes correlated with a shorter patient survival [97]. HTLV-1 and HTLV-2 tropism and spread were also studied by *Kannian et al*. in a rabbit animal model. Results showed that HTLV-2 proviral loads were lower than HTLV-1, and infection was prevalent in CD8+ cells [98].

### Effects of HTLV-1 proteins on the DNA damage response

*Boxus et al*. showed that Tax associates with the minichromosome maintenance MCM2-7 helicase complex and localizes to origins of replication. Through this interaction, Tax fires supplementary origins at the onset of S phase, thus accelerating S phase progression, but also generating replicative stress and activation of the DNA damage response [99]; this pathway is also engaged by HTLV-1 p30 through its ability to bind ATM and REGγ [100], and by HBZ, which increases the sensitivity of Jurkat cells to cell death induced by DNA damaging drugs [101].

### Post-transcriptional effects of Tax

Effects of Tax at the post-transcriptional level are also emerging: *Mocquet et al*. demonstrated that Tax interacts with INT6, sequestering it from the Nonsense Mediated mRNA Decay (NMD) factors UPF1/2. Tax also increases the size and number of P bodies, the site of UPF1 accumulation with mRNAs targeted by NMD. These data suggest that Tax regulates mRNA degradation through NMD [102]. *Van Duyne et al*. reported a down regulation of the RNAi component Drosha in HTLV-1 infected cells; the colocalisation of Tax with Drosha in nuclear speckles suggests functional interactions of Tax with the RNAi machinery [103].

### Complementary-strand genes of HTLV-1 and HTLV-2

*Borowiak et al*. showed that primary leukemic cells isolated from ATLL patients express high amounts of hTERT, the catalytic subunit of the telomerase complex, and exhibit elevated expression of HBZ and Menin, an inhibitor of hTERT expression [104]. *Douceron et al*. observed that expression of HTLV-2 APH-2 and Tax are correlated with HTLV-2 proviral load, but not with lymphocytosis; consistent with this, APH2 (contrary to HBZ of HTLV-1) did not promote cell proliferation *in vitro *[105]. The nuclear localization of complementary strand transcripts of both HTLV-1 and HTLV-2 [95] might favour viral persistence by reducing translation and exposure of the infected cell to the CTL response while allowing function as a non-coding transcripts.

## Immunology

It is now accepted that an efficient CD8^+ ^cytotoxic T cell (CTL) response to HTLV-1 reduces the proviral load and the risk of inflammatory diseases such as HAM/TSP, but it remains possible that CD8^+ ^T cells also contribute to the tissue damage that leads to neurological symptoms. It is also unknown, although it seems highly probable, whether an efficient anti-HTLV-1 CTL response also protects against ATLL.

### CTL response

The Tax protein of HTLV-1 remains a focus of much work on the CTL response, because the frequency of Tax-specific CTLs is higher than that of CTLs specific to any HTLV-1 antigen: that is, the Tax protein is immunodominant in the CTL response. *Kubota et al*. found that HLA-A24-restricted CTLs can recognize low concentrations of Tax, and they suggest that such cells might contribute to the pathogenesis of HAM/TSP [106]. The expression of surface molecules associated with T cell exhaustion (PD-1; Tim-3) is low on HTLV-1-specific CTLs which may help to preserve a functional antiviral response [107,108]. However, it is now apparent that the protective CTL response to HTLV-1 may not in fact be directed against Tax, but against other HTLV-1 proteins. Although the Tax protein is immunodominant, and HLA-A2 is protective in HTLV-1 infection in southern Japan, and Tax peptides can bind strongly to HLA-A2, there is no direct evidence that the strong anti-Tax CTL response itself is protective. Indeed, recent evidence shows that it is efficient CTL recognition of the HBZ protein, not Tax, that confers protection in HTLV-1 infection [109].

### Interferon, interleukins and chemokines

*Kannagi et al*. reported the potentially important finding that type 1 interferon (IFN) can reduce HTLV-1 Gag protein expression *in vitro *[110]. If this is true *in vivo*, it provides an attractive explanation for the puzzling observation that HTLV-1 proviral expression spontaneously and dramatically rises in freshly isolated PBMCs. Proviral transcription may be suppressed *in vivo *by type 1 IFN produced by, for example lymphatic endothelium; this repression would then be released when the cells are incubated *ex vivo*, away from the endothelium. Type 1 IFN has been tried as a treatment for HAM/TSP, albeit with limited success. *Moens et al*. reported that ascorbic acid has a more powerful anti-viral and anti-proliferative effects than IFN-α alpha on freshly isolated PBMCs from HTLV-1-infected people [111]. Just as the CTL response might exert detrimental effects as well as the observed protective effects in HTLV-1 infection, so IFNs may exert mixed effects. *Tattermusch et al*. used gene expression microarray analysis and discovered an IFN signature in HTLV-1 infection: high level expression of IFN-stimulated genes was associated with the presence of HAM/TSP, independently of the proviral load [112]. Perhaps a local excess of both IFNs and CTL activity in the CNS is detrimental, although each is beneficial in the host as a whole. The role of chemokines and cytokines other than IFNs deserves more attention in HTLV-1 infection. IL-15, which promotes replication and survival of NK cells and CD8^+ ^T cells (CTLs), is upregulated in HTLV-1 infection. *Rauch et al*. provided a warning, however, that IL-15 can also promote tumour growth in Tax-transgenic mice [46]. IL-18 is a cytokine that stimulates the production of the potentially inflammatory IFN-γ by NK cells and T cells. *Wagatsuma et al*. reported that certain polymorphisms in the IL-18 gene promoter were more frequent in patients with HTLV-1 infection than uninfected controls in Brazil [113]. However, there was no significant difference in allele frequency between asymptomatic HTLV-1 carriers and patients with HAM/TSP, and it remains in question whether IL-18 plays a role in the pathogenesis of HAM/TSP. HTLV-1 Tax protein has previously been shown to cause infected T cells to secrete the chemokine CCL22, which maintains the high frequency of CD4^+ ^FoxP3^+ ^cells observed in HTLV-1 infection. *Barrios et al*. provided evidence that recombinant Tax from HTLV-1 (Tax-1) and HTLV-2 (Tax-2) can stimulate production of 3 CC chemokines by PBMCs in vitro [114]. Further work is needed to assess the possible significance of this observation in vivo.

### HBZ

It is now realized that the HBZ gene, encoded on the negative strand of the HTLV-1 provirus, plays a critical role in several aspects of HTLV-1 biology. Its importance as the antigen recognized by the protective class 1 HLA-associated immune response was mentioned above. HBZ itself may also counter the immune response: *Douville et al*. found that HBZ may inhibit IFN type 1 responses when transfected into human PBMCs and astrocytes, by down regulating IRF3-dependent signalling [115]. *Miyazato et al*. reported that HBZ both upregulated expression of the transcription factor FoxP3, which is characteristic of regulatory T cells (T_regs_), and simultaneously diminished the suppressive (regulatory) functions of the FoxP3^+ ^cells [116]. Intriguingly, both of these effects have also been reported to be exerted by Tax. *Sugata et al*. observed that HBZ can also repress transcription from the IFN gamma promoter, and the authors suggested that this repression might contribute to the severe immunosuppression characteristic of adult T cell leukaemia/lymphoma [117].

### Antibody response

The antibody response to HTLV-1 has been much less studied than the T cell response. It has long been known that anti-HTLV-antibody can reach very high titres, especially in patients with HAM/TSP. *Akahata et al*. have devised a luciferase immune-precipitation system to quantify anti-HTLV-1 antibodies, and suggest that the assay may be useful as a prognostic or diagnostic test [118]. *Kuo et al*. used recombinant Env proteins to generate potently antiviral monoclonal antibodies in mice [119]. This system may prove useful to identify potentially protective antibody epitopes in Env protein, which varies in sequence compared with the Env protein of HIV-1.

### Future directions

The findings summarized above raise many important questions that deserve more attention in future research. Among these questions, we identify four of particular interest: i) Since HTLV-1 with the same sequence causes the full range of malignant and inflammatory diseases, the outcome of infection must be determined by variation among hosts. The class 1-restricted CTL response accounts for about 50% of the observed risk of HAM/TSP: what accounts for the remaining 50%? And does HLA class 1 genotype also determine the risk of ATLL? ii) How does KIR genotype determine the efficiency of HLA class 1-mediated protection? iii) What is the balance of beneficial and harmful effects of interferon in HTLV-1 infection? What are the causes and consequences of the observed reduction in the number and activity of NK cells in HTLV-1 infection?

## Endogenous retroviruses, foamy viruses and XMRV

The growing importance of non-HTLV human retroviruses was highlighted by the Scientific Committee organizing two sessions entitled "Endogenous retrovirus, foamy viruses and XMRV". Debate and discussion of whether Xenotropic Murine leukemia virus (MLV) Related Virus (XMRV) represents genuine human viruses or laboratory artefacts continued during these two sessions.

### XMRV

Two presentations did not find XMRV or related viruses in persons with chronic fatigue syndrome (CFS) or multiple sclerosis (MS) while a third report found only polytropic MLV in both CFS patients and healthy controls [120-123]. However, two articles published in Science the week of the conference helped elucidate the origin and significance of XMRV. *Propotka et al*. provided strong evidence that XMRV was not present in the human prostate cancer cell line 22Rv1, but rather is a novel mouse gammaretrovirus generated via recombination during the passage of human prostate tissue xenografts in nude mice, containing near perfect chimeras of the 5' and 3' halves of the consensus XMRV genome [124]. *Knox et al*. using serology, culture, and PCR methods, failed to confirm XMRV infection in persons with chronic fatigue syndrome (CFS) [125], including those found positive in the original study by Lombardi *et al*. [126]. *Knox et al*. showed that sera containing complement from CFS patients and matched controls both inactivated XMRV and MLV *in vitro*, restricting infection of human cells with these viruses, suggesting that sustained infection in humans is highly unlikely [125]. They reported evidence of contamination of mouse monoclonal antibodies used in hot start PCR enzymes and cell sorting/staining. Contamination and non-specific serologic reactivity, as sources of false positive results in XMRV studies, were further emphasized in presentations by *Erlwein et al*.[127] and Qiu *et al*. [128]. Qiagen columns used for DNA extraction from paraffin-embedded tissues contained both MLV and XMRV *gag *sequences, adding to the plethora of reagents, such as PCR enzymes, monoclonal antibodies and cell lines found to be contaminated with these viruses and sequences. *Qiu et al*. presented data showing cross-reactivity of sera from HTLV-1-infected persons with a conserved peptide region in the XMRV p15E envelope surface protein. They also reported an absence of reactivity in a large number of US blood donors and HIV-1-infected Africans using well-validated assays, suggesting that previous sero-reactive results obtained by others may be due to cross-reactivity or non-specific binding using incompletely validated tests [128].

Combined with the majority of the negative reports, these findings have questioned the findings from laboratories previously reporting positive results which now needs to exclude the possibility that the positive findings may have arisen from contamination. Such was the case in the presentation by *Hanson et al*. who described the extensive testing done and still to be performed to rule out false positive MLV results in the persons with CFS and healthy controls in their study [120]. Although the field is now heavily favouring an end to the investigation of XMRV and MLV as human viruses, two ongoing studies at multiple institutions, one led by the National Heart, Lung, and Blood Institute (NHLBI) of the NIH as presented by Graham Simmons [129] and one led by Ian Lipkin at Columbia University, should finally clarify whether XMRV and MLV are present in blood donors and persons with CFS, respectively.

### Simian foamy viruses

While the end is seemingly near for XMRV, much more research is needed to better understand the public health importance of human infection with other retroviruses, including simian foamy virus (SFV), a common infection in nonhuman primates (NHPs) [130]. From the meeting, it is clear that SFV is widely distributed across central Africa (Cameroon, Democratic Republic of Congo, Gabon), mostly in persons with exposure to NHPs, but also in persons without reported NHP exposure [123,131,132]. For example, the wife of an SFV-infected hunter in Cameroon was reported to be seropositive, raising the possibility of person-to-person transmission [131]. However, additional work is needed to determine if SFV is transmitted secondarily and if so, at what frequency.

### STLVs

In addition to SFV, other simian retroviruses are known to cross species and infect humans [133]. For example, *Calvignac-Spencer et al*. provided evidence for zoonotic transmission of Simian T-Lymphotropic Virus type 1 (STLV-1) from Colobus monkeys and chimpanzees in Côte d'Ivoire [134]. In contrast, the lack of a closely related STLV has been an enigma for understanding the evolutionary history and origin of HTLV-2. STLV-2 in captive and wild bonobos is genetically similar, but very distinct from HTLV-2 [135]. The finding of only HTLV-2 subtype b strains in Baka Pygmies, some of the oldest inhabitants of Cameroon, phylogenetically related to HTLV-2b in Amerindians, suggests an African origin for both STLV-2 and HTLV-2. HTLV-2b may have co-migrated with the ancient movement of Africans through Asia, across the Bering Strait, and into the Americas [136]. Alternatively, HTLV-2b in the Baka may be due to a more recent introduction into Africa. An expanded search for STLV-2-like viruses in NHPs and testing of other ancient African and Asian populations will help clarify these possibilities.

### Endogenous retroviruses

ERVs integrated into the human genome millions of years ago and have been associated with a variety of illnesses, including cancer and multiple sclerosis (MS), though these associations remain controversial [137]. At the conference, several presentations [138-141] addressed the role of ERVs in MS and various cancers. However, case-control studies and animal model studies using infectious molecular clones are required to fully understand what role, if any, ERVs may play in human disease.

### Future directions

Screening people for retroviruses in conjunction with other blood borne and sexually transmitted infections in endemic areas would help to determine if these viruses are entering the general population and if co-infections facilitate such transmission. Longitudinal, long-term follow-up studies will determine the pathogenicity of these viruses in humans. Establishing the prevalence of so called "harmless" retroviruses, such as SFV, in specific patient cohorts with common chronic conditions, malignant and inflammatory, in endemic regions, would add to our understanding of how these viruses might be contributing to human diseases. Genetic characterization of complete genomes and studying the intra-host and inter-host evolution of these viruses, coupled with animal model studies, will help understand the possible pathogenicity of these novel human viruses. Together, population-based and expanded molecular epidemiologic studies will determine how widespread these viruses are and determine their natural history in their non-human and human primate hosts.

## Conclusions

As anticipated, the 15^th ^HTLV Conference on Human Retrovirology, HTLV and Related Viruses led to the early distribution of exciting new data, especially in the field of molecular and animal models of science. Although little progress had been made specifically in the development of treatment for lymphomatous ATL and HAM/TSP since the 14^th ^Conference in Bahia, Brazil, this conference supported the formation of two clinical trial groups: HAM/TSP and ATL Clinical Trial Subgroups. The aims of these groups are three fold: i) to inform members of relevant published clinical data; ii) to introduce working groups planning future clinical trials and iii) to inform members of ongoing clinical trials and expand recruitment. The HAM/TSP CTSG is currently developing a multicentre, international phase III clinical trial in patients with active HAM/TSP, with principle investigators from Brazil, Japan, Peru, USA and UK. These groups and much more are accessible through the HTLV association website (http://htlv.net). The HTLV 16^th ^conference will be held in Montreal, Canada in June 2013.

## Conflict of interests

None of the authors declared any conflict of interests. Use of trade names is for identification only and does not imply endorsement by the U.S. Department of Health and Human Services, the Public Health Service, or the Centres for Disease Control and Prevention. The findings and conclusions in this report are those of the author and do not necessarily represent the views of the Centers for Disease Control and Prevention. RM is supported by ENS Lyon, INSERM and InCa.

## Authors' contributions

FM wrote the clinical research section; CRMB wrote the immunology section; VC wrote the virology section; MDL wrote the animal models section; ELM wrote the epidemiology section; WMS wrote the endogenous retroviruses foamy viruses and XMRV section; RM wrote the molecular and cellular biology section. FM and RM edited the manuscript. All authors read and approved the final manuscript.
